# New composite nitrite-free and low-nitrite meat-curing systems using natural colorants

**DOI:** 10.1002/fsn3.57

**Published:** 2013-08-22

**Authors:** Mohammad H Eskandari, Sara Hosseinpour, GholamReza Mesbahi, Shahram Shekarforoush

**Affiliations:** 1 Department of Food Science and Technology, College of Agriculture, Shiraz UniversityShiraz, 7144165186, Iran; 2 Department of Food Hygiene, School of Veterinary Medicine, Shiraz UniversityShiraz, 7144165186, Iran

**Keywords:** Cochineal, nitrite free, sausage, sodium hypophosphite

## Abstract

Nitrite-free and low-nitrite meat-curing systems were developed to eliminate or reduce nitrite in frankfurter-type sausages. Different composite meat-curing mixtures were formulated using cochineal and paprika as natural colorants, sodium hypophosphite (SHP) as antimicrobial agent, butylated hydroxyanisole (BHA) as antioxidant and sodium nitrite. The treatment, which contained 0.015% cochineal, most closely resembled the 120 ppm NaNO_2_ in its ability to create cured-meat color. BHA was found to be a strong antioxidant at the 30 ppm level in cooked sausages during refrigerated storage for 5 weeks. All treatments containing 40 ppm sodium nitrite were successful in replicating sensory attributes of frankfurter samples. Our findings support the use of SHP as possible antibotulinal agent in nitrite-free meat-curing systems.

## Practical Applications

Several nitrite-free and low-nitrite frankfurters consisting of natural colorants, an antioxidant and sodium hypophosphite as antimicrobial agent were formulated in this study. The data from this investigation support the use of cochineal as a colorant, sodium hypophosphite as an antibotulinal and antimicrobial agent, and butylated hydroxyanisole (BHA) as an antioxidant in developing of composite nitrite-free and low-nitrite frankfurter.

## Introduction

Meat curing is the application of salt, sugar, nitrite, or nitrate to meat in order to impart unique properties to the end product (Forest et al. 1975; Pegg and Shahidi 2000). Of these, nitrite is the key ingredient and plays a multifunctional role (Shahidi et al. 1985). From a consumer acceptance aspect, the potency of nitrite to develop the pink color of cured-meat products and influence the customers is of paramount importance, but from a health perspective, its ability to prevent the germination of spores of *Clostridium botulinum* is the pivotal role of nitrite (Pegg and Shahidi 2000). Another major function of nitrite in meat curing is its effect on retarding lipid autoxidation and warmed over flavor development (Morrissey and Tichivangana 1985).

Despite all its desirable effects, the addition of nitrite to meat has become a source of controversy in the last four decades. Under certain conditions (e.g., high temperature), residual nitrite may react with free amino acids and amines and form *N*-nitrosamines at trace quantities (ppb scale) in cured-meat products or in stomach after consumption (Huang et al. 1996). Experimental animal studies and epidemiological evidence have shown *N*-nitrosamines to be potent carcinogens (Pegg and Shahidi 2000; Honikel 2008). As a result of increasing concern about *N*-nitrosamines, several approaches have been considered by researchers to reduce exposure to nitrosamines. These include inhibition of nitrosamines formation by ascorbic acid, erythorbic acid, and tocopherol; decrease or elimination of nitrite from curing systems (Gray et al. 1982) and natural curing of meat (Sebranek and Bacus 2007).

As nitrite is responsible for imparting several desired properties to cured meats, it seems that the possibility of finding a single compound to perform all the functions of nitrite is quite dim (Shahidi and Pegg 1992). Therefore, much effort had concentrated on the preparation of multicomponent curing systems to provide different roles of nitrite. Such composite systems consist of a colorant, an antioxidant, and an antimicrobial agent. Numerous studies have evaluated the potential application of nitrite alternatives in nitrite-free meat-curing systems (Pegg and Shahidi 2000), but there is limited investigation into the simultaneous substitution of nitrite roles in a cured-meat product. Very often, various semisynthetic and synthetic food additives can be used to mimic nitrite roles in nitrite-free meat-curing systems (Shahidi 1991).

Preformed cooked cured-meat pigment (PCCMP) has been used successfully to reproduce attractive color in nitrite-free curing systems. However, preparation of PCCMP is laborious and time consuming. PCCMP is also sensitive to light and oxygen and there are questions regarding its safety (Shahidi and Pegg 1992; Corpet 2011). Natural colorants have become increasingly popular with consumers, and their ability to improve the color of meat products has been attempted (Bloukas et al. 1999). Cochineal is the red color, which is obtained from an insect (*Dactilopious coccus*), and paprika is obtained from dried pods of sweet pepper (Delgado-Vargas and Paredes-López 2002). Both colors could be used to mimic the cured-meat color.

Therefore, the first objective of this study was to use cochineal and paprika in combination with other curing alternatives to mimic curing color in nitrite-free and low-nitrite multicomponent systems for the commercial production of frankfurter-type sausages.

The antibotulinal activity of several substances has been studied in emulsion-type meat products, but most of these compounds were shown to have ineffective properties when compared with a control manufactured with 120 mg/kg (Sindelar and Houser 2009). Sodium hypophosphite (SHP), a generally recognized as safe (GRAS) substance, has been used effectively as an antibotulinal agent in meat slurries (Wood et al. 1986) and bacon (Pegg and Shahidi 2000). The second objective of this research was to evaluate antibotulinal activity of SHP in composite nitrite-free and low-nitrite frankfurter sausages. Eighteen different frankfurter treatments (TRTs) were prepared using cochineal and paprika as colorants, SHP as antimicrobial agent, BHA as antioxidant, and sodium nitrite, and the quality and safety of samples were assessed during 8 weeks of storage.

## Material and Methods

### Preparation of spore suspension of *Clostridum botulinum*

The spore inoculum was a mixture of two strains of *C. botulinum,* type A (SU-1345) and nonproteolytic type B (SU-1373) of soil origin. Spore suspensions were prepared according to Smith (1975). Tubes of 10 mL cooked-meat broth (Merck, Darmstadt, Germany) were inoculated with frozen spore suspensions. After 24 h of anaerobic incubation at 30°C, tryptone peptone glucose yeast extract (TPGY) agar media were inoculated anaerobically at 35°C for 5 days.

The spores, harvested by scraping the agar surface, were washed three times in normal saline and stored at −20°C until they were used. The enumeration of spores was performed with a Petroff-Hauser hemocytometer and also after plating on TPGY agar medium (DIN 1988). The composite spore suspensions were heat shocked (80°C for 15 min to produce toxin-free spore suspension), appropriately diluted, and added to the meat batter during formulation for treatments to be evaluated for *C. botulinum* inhibition (TRTs 14–18).

### Ingredients

Commercial frozen, boneless beef cuts from rump and topside were obtained from a local meat market. Partially thawed beef was ground through an 8 mm plate. Sodium nitrite, SHP, and BHA were purchased from sigma (Sigma–Aldrich Ltd., St. Louis, MO). Carmine (50% w/w in coloring material) and paprika oleoresin (40,000 coloring unit per gram) were purchased from Chr. Hansen (Chr. Hansen's Laboratorium Denmark A/S, Horsholm, Denmark). Paprika powder (*Capsicum annuum*) was purchased from a local market.

### Preparation of frankfurters

All TRTs, about 5 kg each, were replicated from a separate meat source as three different preparations. The basic formulation used for frankfurters was derived from local meat factories. The following raw materials and ingredients were used per kg of batter: beef meat 600 g, sunflower oil 80 g, ice/water 180 g, wheat starch 40 g, soybean flour 40 g, salt 16 g, garlic powder 4 g, sodium ascorbate 0.4 g, sodium polyphosphate 4 g, and seasoning 7 g. Other ingredients were added as required for each treatment based on Table 1.

**Table 1:** Treatment formulation for production of nitrite-free and low-nitrite sausages

Treatments	Additives
1.	NaNO_2_ (120 ppm)
2.	NaNO_2_ (40 ppm)
3.	No additive
4.	NaNO_2_ (40 ppm) + sodium hypophosphite (1000 ppm) + paprika extract (0.1% v/w)
5.	NaNO_2_ (40 ppm) + sodium hypophosphite (1000 ppm) + cochineal powder (0.002% w/w)
6.	Sodium hypophosphite (3000 ppm) + paprika powder (1% w/w)
7.	Sodium hypophosphite (3000 ppm) + paprika extract (0.1% v/w)
8.	Butylated hydroxyl anisole (30 ppm)
9.	NaNO_2_ (40 ppm) + butylated hydroxyl anisole (30 ppm)
10.	Paprika powder (0.1% w/w)
11.	Cochineal powder (0.02% w/w)
12.	Cochineal powder (0.015% w/w)
13.	Paprika extract (1.5% v/w)
14.	2 × 10^3^ Spores *Clostridium botulinum*/g + NaNO_2_ (120 ppm)
15.	2 × 10^3^ Spores *C. botulinum*/g + NaNO_2_ (40 ppm)
16.	2 × 10^3^ Spores *C. botulinum*/g
17.	2 × 10^3^ Spores *C. botulinum*/g + NaNO_2_ (40 ppm) + sodium hypophosphite (1000 ppm)
18.	2 × 10^3^ Spores *C. botulinum*/g + sodium hypophosphite (3000 ppm)

**Figure 1:**
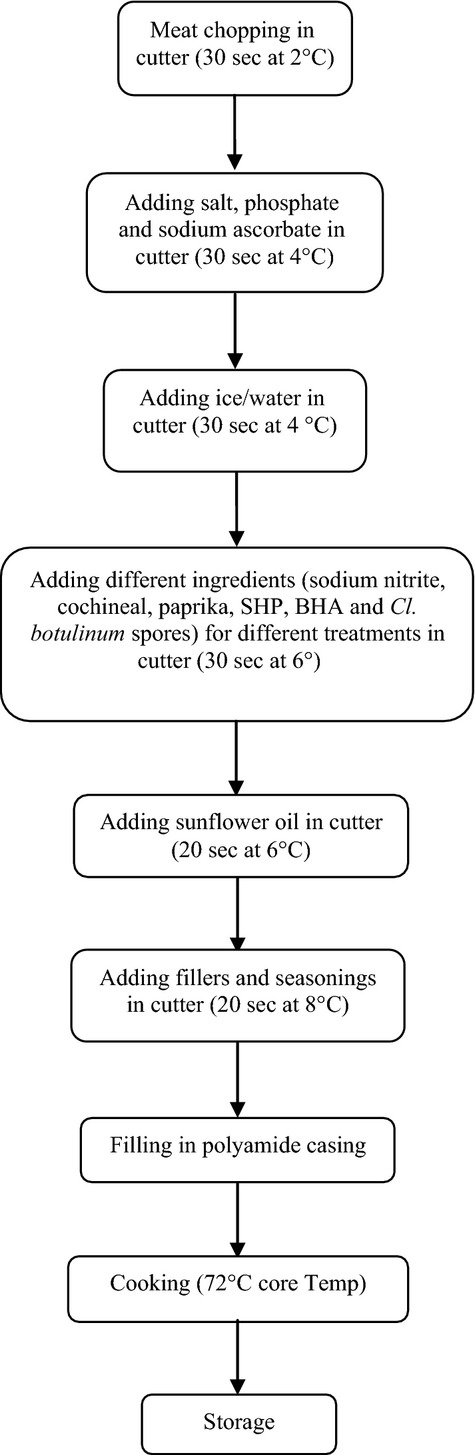
Sausage production flow diagram. SHP, sodium hypophosphite; BHA, butylated hydroxyanisole.

Partially thawed beef was chopped in an Alexanderwerk 15-L cutter (Alexanderwerk, Remscheid, Germany) at low speed for 15 sec and mixed with the salt, phosphate, and sodium ascorbate. The mass was dry chopped for 20 sec at high speed. Half of the ice/water was added and the batter was chopped for 1 min (Fig. 1). The sunflower oil was gradually added. The remaining half of ice/water and other remaining ingredients were added and chopped until the meat emulsion reached 14°C. Eighteen treatments of frankfurters (Table 1) were formulated based on previous research findings (Wood et al. 1986; Bloukas et al. 1999) with three levels of sodium nitrite (0, 40, and 120 mg/kg) and two levels of SHP (1000 and 3000 mg/kg) as antimicrobials; three levels of cochineal (0.002%, 0.015%, and 0.02%), two levels of paprika extract (1 and 15 mL/kg), 1% of paprika powder as natural colorants; and 30 mg/kg of BHA as antioxidant. Thirteen TRTs were used for chemical, physical, and sensory analysis. For five TRTs, spores of *C. botulinum* (2 × 10^3^/g of batter) were added to the batter using a disposable mixer. These products were stored at 4 and 10°C for 8 weeks and evaluated for microbial analysis. Immediately after chopping, the samples were taken for chemical and physical analysis. The emulsion was stuffed with an Alexanderwerk 15-L stuffer (Alexanderwerk) into 24-mm-diameter polyamide casings. Sausages were hand linked at 12 cm intervals, showered, and cooked in water until the core temperature reached 72°C.

After heat processing, the frankfurters were cooled under a cold water shower and stored at 4°C for subsequent analysis. A part of frankfurter samples containing spores of *C. botulinum* was vacuum packed and held at 10 and 25°C to investigate the effects of abuse temperature on botulinum toxin production.

### Proximate analysis

The moisture content of cooked frankfurters (all TRTs) was determined by oven drying at 104°C for a period of 16 h. Protein content was measured by the Kjeldahl method (Association of Official Analytical Chemists, [AOAC] 2000a) and the fat content of samples was determined by the soxhlet extraction method (Association of Official Analytical Chemists, [AOAC] 2000b). The ash content was determined according to the standard AOAC procedures (Association of Official Analytical Chemists [AOAC] 2000c). Three replicates per treatments were analyzed.

### pH value

The pH values of sausage batters and frankfurters (all TRTs) were determined in a homogenate prepared with 10 g of samples and 90 mL of distilled water, using a Crison 2001 pH meter (Crison Instruments SA, Barcelona, Spain).

### Residual nitrite analysis

Residual nitrite was determined by the AOAC (2000d) method in raw batter and sausage samples (TRTs 1, 2, 5, 8, and 9). At the same time, all treatments were analyzed to minimize variation in the analysis due to time.

### Measurement of lipid oxidation

Lipid oxidation was monitored in cooked sausages (TRTs 1, 2, 3, 8, and 9) during storage by the thiobarbituric acid reactive substance method according to Botsoglou et al. (1994) and Ulu (2004).

A 2-g sample was placed in a 50-mL test tube and homogenized with 8 mL of 5% trichloro acetic acid (TCA) and 5 mL of 0.8% butylated hydroxyl toluene (BHT) in hexane. The homogenate was centrifuged for 5 min at 4000*g* and the middle aqueous layer was filtered. Two milliliters of filtrate was pipette into a screw-capped tube to which 1.5 mL of 0.8% aqueous TBA and 0.5 mL of 5% TCA were added. Following incubation for 40 min at 80°C, the tube was cooled under tap water and the absorbance was measured by a UV scanning spectrophotometer (MSE Scientific Instruments, Crawley, Sussex, U.K.) at 532 nm against a blank containing 5-mL distilled water and 5 mL of 0.8% aqueous TBA. The results were expressed as mg malondialdehyde (MDA) per kg of raw batter or cooked sausages.

### Microbiological analysis

Bacteriological evaluation assay was undertaken prior to sensory analysis. For each frankfurter sample (TRTs 1, 2, 3, 4, 5, 6, and 8), 10 g was taken and placed in a sterile plastic bag with 90 mL of sterile normal saline. After 2 min in a stomacher (Model BA 6024; A. J. Seward and Company Ltd, London, U.K.), appropriate decimal dilutions were pour plated on the following media: Plate Count Agar (PCA) (Merck) for total mesophilic aerobic count (TMAC) (30°C for 48 h); De Man, Rogosa, Sharp Agar (MRS; Merck) for lactic acid bacteria count (anaerobically, 32°C for 48 h); violet red bile agar (VRBA) (Merck) for coliform count (37°C for 24 h); and potato dextrose agar (PDA; Merck) for yeast and mold count (30°C for 5 days).

Duplicate analyses were conducted for each treatment and the results were expressed as 1og CFU/g frankfurter.

### Toxin assay

Toxin assay was undertaken prior to sensory analysis. In this session, TRTs 14–18 were used for analysis. Generally, the mouse bioassay followed Wood et al. (1986) and German DIN (1988) guideline. Twenty grams from each sausage pack was removed and homogenized with 20 mL of gelatin phosphate buffer (0.2% gelatin, 0.4% disodium hydrogen phosphate, pH 6.2) using a stomacher for 2 min. The homogenate was centrifuged at 8000*g* for 5 min at 4°C.

The supernatant fluid was removed and filtered through a 45-μm membrane filter. 0.5 mL of filter sterilized supernatant fluid was injected interperitoneally into three mice. The animals were observed and clinical signs or deaths were reported over a period of 4 days.

### Sensory analysis

A 10-member trained panel evaluated frankfurter samples (TRTs 1–13) for their color, aroma, taste, texture, and overall acceptance according to the 7-point hedonic scale (7 = extremely like; 1 = extremely dislike). Sensory analysis was performed by staff of the Department of Food Science and Technology. Samples were cut into 3 cm sections, reheated to 45°C in a microwave, and presented to panelists with three-digit code and in random order. Water was provided for rinsing between samples. Testing was conducted in partitioned booths and under incandescent/fluorescent light for the color evaluation.

### Color measurement

The surface color of frankfurter samples (TRTs 1–13) was evaluated using a method of Yam and Papadakis (2004). A Canon digital camera (Canon Ixus 120 IS; Canon, Tokyo, Japan), with a resolution of 300 dpi, was installed at a constant distance (30 cm) from the batter or sausage surface and was used for taking digital images. The lamp and the camera were placed in a box (50 × 50 × 60 cm) with interior white color walls. The angle between the sausage surface and the axis of the camera lens was 90° and the angle between sausage surface and light source was 45°C. Illumination was achieved using a 60-W fluorescent light lamp (Natural Day Light; Cixing, Beijing, China). The digital images of samples were saved in JPEG format and analyzed in the lab mode to obtain *L*, *a,* and *b* color parameters using Adobe Photoshop 8.0 software (Adobe, San Jose, CA).

### Statistical analysis

Three independent replicates of each experimental treatment were carried out at 2-month intervals. All data were analyzed by one-way analysis of variance using SPSS, version 11.5 software (SPSS, Chicago, IL), which was followed by Duncan's multiple range test. A *P* < 0.05 was considered statistically significant.

## Results and Discussion

### Proximate analysis and pH

Proximate analysis of moisture, protein, fat, and ash was determined for frankfurter treatments. The average moisture content of frankfurter sausage was 67.2% and ranged from 66.4% to 68.1%. The average protein content was 16.35% and ranged from 16.3% to 16.4%. The average fat content was 11.1% and ranged from 10.6% to 11.5%, and finally, the average ash content was 2.98% and ranged from 2.90% to 3.07%. These data show that frankfurter treatments were uniform in proximate composition. No significant difference (*P* > 0.05) was found in the proximate composition between frankfurter TRTs.

The pH value of sausage batters ranged from 6.0 to 6.1. The pH of frankfurters was increased by nearly 0.1–0.2 pH unit during heat processing. The same increase in pH during heat treatment was reported by Ruusunen et al. (2003). The pH of cooked frankfurters ranged from 6.15 to 6.30. No significant difference (*P* > 0.05) was found between the pH values of cooked sausages. Also, the pH values for cooked frankfurters were very similar. The pH values for cooked sausages were decreased slightly during refrigerated storage (4°C) for 8 weeks (data not shown), but only the decrease in pH value was significant for frankfurter with 0 ppm of sodium nitrite (TRT 3), ranging from 6.21 to 6.03 after 8 weeks of storage at 4°C. The decrease in pH may be due to growth and activity of bacteria which ferment the carbohydrates (Candogan and Kolsarici 2003).

### Residual nitrite

**Figure 2:**
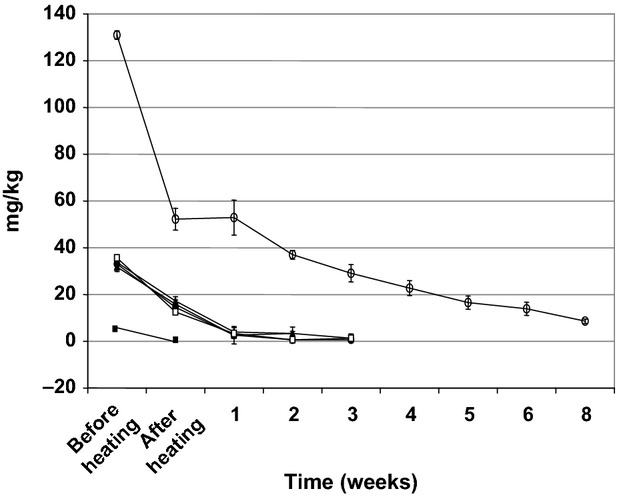
Residual nitrite (ppm) in raw batters and frankfurter samples during storage at 4°C. All values are mean ± standard deviation. ○: NaNO_2_ (120 ppm), (TRT 1); •: NaNO_2_ (40 ppm), (TRT 2); △:NaNO_2_ (40 ppm) + sodium hypophosphite (1000 ppm) + paprika extract (0.1% v/w), (TRT 4); ▲: NaNO_2_ (40 ppm) + sodium hypophosphite (1000 ppm) + cochineal powder (0.002% w/w), (TRT 5); ▪: butylated hydroxyanisole (30 ppm), (TRT 8); □: NaNO_2_ (40 ppm) + butylated hydroxyanisole (30 ppm), (TRT 9).

The values of residual nitrite are demonstrated in Figure 2. As expected, residual nitrite concentrations were affected by thermal processing and subsequent refrigerated storage in all treatments. Depending on the formulation, 35–50% of the initially added NaNO_2_ was detected as residual nitrite 1 day after heat treatment. Many studies have demonstrated that the added nitrite is rapidly disappeared in meat products as nitrite reacts with meat constituents (Deda et al. 2007; Honikel 2008). No detectable amount of residual nitrite was found in nitrite free (TRT 8 with 0 ppm NaNO_2_ and 30 ppm BHA) and low nitrite (TRTs 2, 4, 5, and 9 with 40 ppm NaNO_2_) 1 day and 3–4 weeks after thermal processing, respectively, whereas residual nitrite was detected in TRT 1 (120 ppm NaNO_2_) by the end of experiments. Nitrite depletion in meat products has healthy effects because it reduces the dietary nitrite intake and the possibility of formation of carcinogenic nitrosamines (Cassens et al. 1979; Ahn et al. 2004). Residual nitrite concentration in a meat product is dependent on a number of factors including the thermal processing, the pH of the product, the addition of reducing agents, the temperature of storage, and the proximate composition of meat product (Honikel 2008; Delgado-Pando et al. 2011). It has been shown that in comparison with 120 ppm sodium nitrite, combination of 40 ppm NaNO_2_ and 2600 mg/kg sorbate reduces *N*-nitrosamine formation in cured products from nearly 100 mg/kg to less than 5 μg/kg (Shaver 1979). Therefore, the application of nitrite-free and low-nitrite meat-curing systems is an effective approach to reduce exposure to nitrosamines.

### Microbiological evaluation and toxin assay

Results of the TMAC of the sausage samples during the 8 week storage period at 4 and 10°C are presented in Tables 2 and 3, respectively.

**Table 2:** Total mesophilic aerobic counts of treated frankfurter sausage (log_10_ cfu/g) during refrigerated storage at 4°C

	Frankfurter formulations				
	
Storage time (weeks)	1	2	3	4	6
0 (day after production)	4.08 ± 0.02^a,A^	4.05 ± 0.01^a,A^	4.17 ± 0.02^b,A^	4.35 ± 0.04^c,C^	4.34 ± 0.01^c,B^
1	4.19 ± 0.02^b,A^	4.12 ± 0.01^a,B^	4.32 ± 0.01^c,B^	4.08 ± 0.02^a,A^	4. 4 ± 0.03^a,BC^
2	4.2 ± 0.04^b,A^	4.18 ± 0.04^b,B^	4.54 ± 0.03^c,C^	4.07 ± 0.04^a,A^	4.16 ± 0.03^b,A^
3	4.2 ± 0.01^b,A^	4.64 ± 0.02^c,C^	4.54 ± 0.01^c,C^	4.07 ± 0.04^a,A^	4.16 ± 0.03^a,B^
4	4.09 ± 0.36^a,A^	4.19 ± 0.07^b,B^	4.71 ± 0.03^d,D^	4.2 ± 0.02^b,B^	4.35 ± 0.02^c,B^
5	4.24 ± 0.02^a,A^	4.64 ± 0.02^c,C^	4.7 ± 0.01^c,D^	4.3 ± 0.03^a,C^	4.49 ± 0.05^b,C^
6	4.08 ± 0.02^a,A^	4.6 ± 0.01^c,C^	4.74 ± 0.00^d,D^	4.07 ± 0.06^a,A^	4.43 ± 0.04^b,BC^
8	4.19 ± 0.02^a,A^	4.65 ± 0.03^c,D^	5.67 ± 0.02^c,E^	4.24 ± 0.01^a,BC^	4.52 ± 0.01^b,D^

All values are mean ± standard deviation. ND, not detectable. 1: NaNO_2_ (120 ppm); 2: NaNO_2_ (40 ppm); 3: no additive; 4: NaNO_2_ (40 ppm) + sodium hypophosphite (1000 ppm) + paprika extract (0.1% v/w); 6: sodium hypophosphite (3000 ppm) + paprika powder (1% w/w).

^A–E^values within each column with different superscripts are significantly different (*P* < 0.05).

^a–c^values within each row with different superscripts are significantly different (*P* < 0.05).

**Table 3:** Total mesophilic aerobic counts of treated frankfurter sausage (log_10_ cfu/g) during refrigerated storage at 10°C

	Frankfurter formulations				
	
Storage time (weeks)	1	2	3	4	6
0 (day after production)	4.32 ± 0.02^c,B^	4.08 ± 0.03^a,A^	4.4 ± 0.06^d,A^	4.32 ± 0.0^c,BC^	4.20 ± 0.73^b,A^
1	4.15 ± 0.02^a,A^	5.65 ± 0.01^d,B^	4.35 ± 0.01^b,A^	4.46 ± 0.01^b,D^	5.02 ± 0.02^c,B^
2	4.25 ± 0.02^a,B^	5.69 ± 0.75^b,B^	4.7 ± 0.01^b,B^	4.18 ± 0.04^a,AB^	4.68 ± 0.03^b,B^
3	4.42 ± 0.01^a,C^	6.17 ± 0.02^d,B^	4.78 ± 0.02^a,B^	4.3 ± 0.06^a,BC^	4.68 ± 0.03^a,B^
4	4.21 ± 0.17^a,B^	6.98 ± 0.03^e,C^	4.69 ± 0.02^c,B^	4.34 ± 0.07^b,C^	5.17 ± 0.02^d,B^
5	4.16 ± 0.17^a,A^	7.16 ± 0.04^e,C^	5.97 ± 0.11^d,C^	4.35 ± 0.04^b,C^	4.97 ± 0.04^c,B^
6	4.11 ± 0.02^a,A^	7.35 ± 0.01^d,CD^	6.48 ± 0.02^c,D^	4.09 ± 0.02^a,A^	5.83 ± 0.04^b,C^
8	5.35 ± 0.01^b,D^	7.98 ± 0.02^e,D^	6.89 ± 0.01^d,D^	4.29 ± 0.03^a,BC^	6.28 ± 0.03^e,C^

All values are mean ± standard deviation. 1: NaNO_2_ (120 ppm); 2: NaNO_2_ (40 ppm); 3: No additive; 4: NaNO_2_ (40 ppm) + sodium hypophosphite (1000 ppm) + paprika extract (0.1% v/w); 6: sodium hypophosphite (3000 ppm) + paprika powder (1% w/w).

^A–D^values within each column with different superscripts are significantly different (*P* < 0.05).

^a–e^values within each row with different superscripts are significantly different (*P* < 0.05).

Initial TMAC of all frankfurter treatments did not differ and ranged from 4.05 to 4.40 log_10_ cfu/g (*P* > 0.05). TMAC of frankfurter samples remained lower than 5 log_10_ cfu/g, during the 8 weeks of storage at 4°C. Significant increase only occurred in TMAC of TRT 3 (0 ppm NaNO_2_) by the end of storage at 4°C (*P* < 0.05). Addition of 1000 mg/kg SHP and 40 mg/kg sodium nitrite (TRT 4) was found to have a significant impact on TMAC of sausage during storage at 10°C (*P* < 0.05) and the TMAC of this treatment remained lower than 5 log_10_ cfu/g up to 8 weeks of storage. Significant increase in TMAC in TRT 1 (120 ppm sodium nitrite) was only observed at the 8 weeks of storage (*P* < 0.05), whereas in the presence of 3000 mg/kg SHP (TRT 6), there was a significant increase in microbial count after 5 weeks of storage at 10°C.

Levels of lactic acid bacteria and yeast and mold counts were lower than 1 log_10_ cfu/g during storage for 8 weeks at 4 and 10°C. Also, no coliforms were detected throughout the 8 weeks of storage due to adequate heat treatment during production. These counts were similar to those reported by other researchers (Candogan and Kolsarici 2003; Lόpez-Lόpez et al. 2009).

All frankfurter samples incubated at 10°C did not develop toxin after 60 days of storage (data not shown). Wood et al. (1986) also reported no toxin production in meat slurries with and without additives after 2 months of refrigerated storage at 5°C.

The first signs of toxin production appeared after 3 days of incubation in sausages with no preservative (TRT 16). Addition of 120 or 40 ppm of sodium nitrite (TRT 14 and TRT 15) prevented toxin production in the sausages during storage at 25°C for 7 days. Sausage samples containing 3000 mg/kg SHP (TRT 18) most closely resembled the frankfurter treatment No. 14 (120 mg/kg NaNO_2_) in its ability to prevent toxin production. Sausages containing 1000 mg/kg SHP and 40 mg/kg NaNO_2_ (TRT 18) became toxic on the fifth day of storage at 27°C. The data from these experiments support the use of SHP as possible antibotulinal agents in nitrite-free meat-curing systems. SHP is bland in taste and has no adverse effect on the oxidative stability or the color of meat products. In addition, it has been shown that SHP is potentially a good antimicrobial food ingredient and it is GRAS (Rhodehamel and Pierson 1990; Pegg and Shahidi 2000).

### Evaluation of lipid oxidation

**Figure 3:**
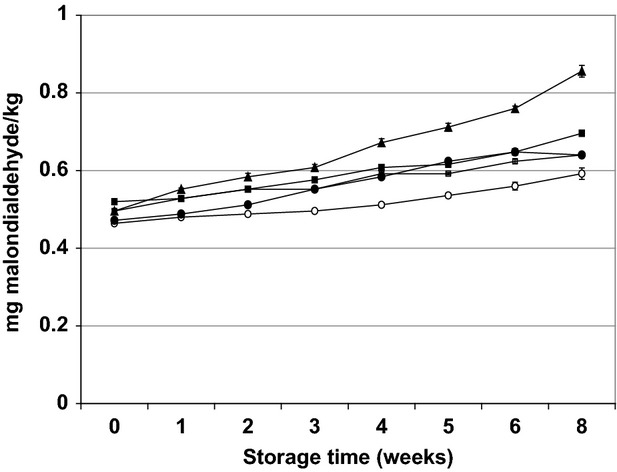
Thiobarbituric acid (TBA) values (mg malondialdehyde/kg sample) of treated frankfurter sausage during storage at 4°C. All values are mean ± standard deviation. ○: NaNO_2_ (120 ppm), (TRT 1); •: NaNO_2_ (40 ppm), (TRT 2); ▲: no additive (TRT 3); ▪: butylated hydroxyanisole (30 ppm), (TRT 8); □: NaNO_2_ (40 ppm) + butylated hydroxyanisole (30 ppm), (TRT 9).

The oxidation level of frankfurter samples, shown in Figure 3, was detected by TBA test for 8 weeks of refrigerated storage at 4°C. As expected, the sample with no added sodium nitrite (TRT 3) had the most lipid oxidation and TRT 1 (120 ppm sodium nitrite) had the lowest TBA value by the end of storage. Sodium nitrite, at both 40 and 120 ppm concentrations, effectively retarded lipid oxidation in cooked sausages and the TBA values were less than 0.7 after 8 weeks of refrigerated storage. According to Shahidi et al. (1987), BHA was found to be a strong antioxidant at the 30 ppm level in cooked sausages over 5 weeks of refrigerated storage. TBA values of TRT 3 (0 ppm sodium nitrite) were higher than those of other frankfurter treatments after day 7 of storage (*P* < 0.05).

### Color evaluation

Natural colorants and their combination with nitrite significantly affected all color attributes of batters and frankfurters (*P* < 0.05) (Tables 4 and 5). TRT 13 (0 mg/kg sodium nitrite + 15 mg/kg paprika oleoresin) had the lowest *L* value and the most *b* value among the batters. Sodium nitrite (120 and 40 mg/kg) significantly decreased the redness of batters (*P* < 0.05). This is due to oxidative action of nitrite and conversion of the red oxygenated myoglobin to brown metmyoglobin (Bloukas et al. 1999). Also, cochineal (TRT 11 and TRT 12) significantly increased the redness of batters (*P* < 0.05).

**Table 4:** Color parameters of different treated frankfurter batters

	Color parameter		
	
Frankfurter formulations	Lightness (L*)	Redness (a*)	Yellowness (b*)
1	58.62 ± 0.87^b^	1.77 ± 0.73^a^	26.38 ± 1.04^b,c^
2	58.38 ± 0.65^b^	5.85 ± 0.8^b^	26.62 ± 1.04^b,c,d^
3	54.69 ± 1.25^a,b^	14.08 ± 0.95^d^	26.38 ± 1.12^b,c^
4	56.46 ± 0.78^a^	6.77 ± 0.73^b^	27.38 ± 1.71^c,d^
5	51.54 ± 1.45^a,b^	10.08 ± 0.76^c^	24.62 ± 1.19^b^
6	56.23 ± 1.17^a,b^	14.77 ± 0.83^d^	28.62 ± 0.87^d,e^
7	54.08 ± 1.75^a,b^	17.62 ± 1.56^e^	27.54 ± 2.22^c,d^
8	55.1 ± 1.25^a,b^	14.17 ± 0.95^d^	26.89 ± 1.15^a^
9	58.73 ± 1.22^a,b^	5.62 ± 1.01^b^	26.42 ± 1.05^a^
10	58.00 ± 0.82^b^	10.46 ± 0.66^c^	26.15 ± 1.34^b,c^
11	53.31 ± 1.11^a,b^	22.92 ± 1.5^f^	19.92 ± 0.86^a^
12	52.36 ± 1.4^a,b^	21.23 ± 1.17^f^	19.38 ± 0.87^a^
13	47.79 ± 1.04^a^	17.00 ± 0.91^e^	30.77 ± 1.36^e^

All values are mean ± standard deviation. 1: NaNO_2_ (120 ppm); 2: NaNO_2_ (40 ppm); 3: No additive; 4: NaNO_2_ (40 ppm) + sodium hypophosphite (1000 ppm) + paprika extract (0.1% v/w); 5: NaNO_2_ (40 ppm) + sodium hypophosphite (1000 ppm) + cochineal powder (0.002% w/w); 6: sodium hypophosphite (3000 ppm) + paprika powder (1% w/w); 7: sodium hypophosphite (3000 ppm) + paprika extract (0.1% v/w); 8: butylated hydroxyl anisole (30 ppm); 9: NaNO_2_ (40 ppm) + butylated hydroxyl anisole (30 ppm); 10: paprika powder (0.1% w/w); 11: cochineal powder (0.02% w/w); 12: cochineal powder (0.015% w/w); 13: paprika extract (1.5% v/w).

^a–f^values within each column with different superscripts are significantly different (*P* < 0.05).

**Table 5:** Color parameters of different treated frankfurter samples

	Color parameter		
	
Frankfurter formulations	Lightness (L*)	Redness (a*)	Yellowness (b*)
1	57.27 ± 1.1^d^	13.2 ± 0.77^d^	19.33 ± 1.4^b,c,d,e^
2	57.4 ± 0.91^d^	12.03 ± 1.16 ^c,d^	18.6 ± 1.4^b,c,d^
3	58.53 ± 0.99^d^	6.47 ± 1.06^a^	21.27 ± 1.1^e^
4	57.6 ± 0.74^d^	12.6 ± 1.12^c,d^	19.33 ± 0.98^b,c,d,e^
5	57.73 ± 0.8^d^	13.27 ± 1.16^d^	18.2 ± 0.93^a,b^
6	57.93 ± 0.59^d^	7.00 ± 1.21^a,b^	23.8 ± 1.42^f^
7	56.8 ± 1.01^d^	7.93 ± 1.3^a,b^	21.53 ± 2.78^c,d,e^
8	58.47 ± 1.3^d^	6.6 ± 0.91^a,b^	20.13 ± 1.41^d,e^
9	57.33 ± 0.9^d^	12.07 ± 0.92^c^	17.6 ± 1.18^b,c^
10	58.07 ± 0.7^d^	8.33 ± 0.82^b^	24.4 ± 0.63^f^
11	51.67 ± 1.45^b^	15.07 ± 1.03^f^	14.8 ± 0.73^a^
12	55.27 ± 0.68^c^	12.2 ± 0.94^c,d^	17.4 ± 0.83^b,c,d^
13	48.73 ± 2.02^a^	7.2 ± 0.94^a,b^	28.00 ± 1.68^g^

All values are mean ± standard deviation. 1: NaNO_2_ (120 ppm); 2: NaNO_2_ (40 ppm); 3: No additive; 4: NaNO_2_ (40 ppm) + sodium hypophosphite (1000 ppm) + paprika extract (0.1% v/w); 5: NaNO_2_ (40 ppm) + sodium hypophosphite (1000 ppm) + cochineal powder (0.002% w/w); 6: sodium hypophosphite (3000 ppm) + paprika powder (1% w/w); 7: sodium hypophosphite (3000 ppm) + paprika extract (0.1% v/w); 8: butylated hydroxyl anisole (30 ppm); 9: NaNO_2_ (40 ppm) + butylated hydroxyl anisole (30 ppm); 10: paprika powder (0.1% w/w); 11: cochineal powder (0.02% w/w); 12: cochineal powder (0.015% w/w); 13: paprika extract (1.5% v/w).

^a–f^values within each column with different superscripts are significantly different (*P* < 0.05).

The heat processing significantly affected all color attributes of frankfurters (*P* < 0.05). The redness of frankfurters containing nitrite (120 and 40 mg/kg) was significantly increased by heat processing (*P* < 0.05) (Table 5). The addition of nitrite to meat was followed by thermal processing and produced a relatively stable pink-colored pigment, nitrite hemochrome (Forest et al. 1975). Frankfurter with 0.02% cochineal (TRT 11) had the most intensive red color. This is in agreement with the results reported by Bloukas et al. (1999) and Stuempel (1997). The treatment, which contained 0.015% cochineal, most closely resembled the 120 ppm NaNO_2_ in its ability to develop cured-meat color. Cochineal is quite stable against light, pH variations, and thermal treatments in meat products (Delgado-Vargas and Paredes-López 2002); therefore, its application as a coloring agent in nitrite-free cured-meat products is recommended.

### Sensory evaluation

Table 6 shows the sensory scores for color, aroma, texture, taste, and overall acceptance. There was no significant difference between the samples in terms of aroma and texture evaluation. For color, frankfurters containing sodium nitrite (120 and 40 mg/kg) had the highest score, although no significant differences were observed between these treatments and samples containing 0.015% of cochineal (*P* > 0.05) (Table 6). Treatments with no added sodium nitrite (TRT 3, TRT 6, TRT 7, TRT 8, TRT 10, and TRT 13) had the lowest scores for color. No significant difference was observed between treatments containing 40 and 120 mg/kg sodium nitrite for overall acceptance. This suggests that all treatments with 40 mg/kg NaNO_2_ were successful in replicating sensory attributes of frankfurters with 120 mg/kg sodium nitrite (*P* > 0.05). For flavor, treatments containing nitrite (40 and 120 mg/kg) had the highest scores and were found in sausages with no added NaNO_2_.

**Table 6:** Sensory scores of different treated frankfurter sausage

	Attribute				
	
Frankfurter formulations	Color	Aroma	Texture	Flavor	Overall acceptance
1	6.13 ± 0.74^e^	6.40 ± 0.51^a^	6.27 ± 0.80^b^	5.53 ± 0.64^d^	6.40 ± 0.51^b^
2	6 ± 0.76^e^	6.13 ± 0.52^a,b^	6.00 ± 0.65^a,b^	5.27 ± 0.80^c,d^	6.20 ± 0.68^b^
3	2.60 ± 1.06^e^	6.27 ± 0.46^a,b^	5.80 ± 0.56^a,b^	4.27 ± 1.03^a^	3.87 ± 0.64^a^
4	6.00 ± 0.53^e^	6.00 ± 0.65^a,b^	6.40 ± 0.74^b^	5.47 ± 1.06^d^	6.27 ± 0.88^b^
5	6.33 ± 0.62^e^	6.20 ± 0.41^a,b^	6.40 ± 0.83^b^	6.67 ± 0.72^d^	6.47 ± 0.52^b^
6	2.80 ± 1.08^a,b^	5.93 ± 0.70^a,b^	5.7 ± 0.83^a,b^	4.67 ± 0.72^a,b^	4.07 ± 0.88^a^
7	3.60 ± 1.06^c^	5.87 ± 0.64^b^	6.07 ± 0.70^b^	4.47 ± 0.74^a,b^	4.27 ± 0.88^a^
8	3.06 ± 0.96^a,b^	6.20 ± 0.86^a,b^	6.07 ± 0.70^a,b^	5.07 ± 0.70^b,c,d^	3.73 ± 0.80^a^
9	5.93 ± 0.59^e^	6.33 ± 0.49^a,b^	6.07 ± 0.59^a,b^	5.33 ± 0.90^d^	6.27 ± 0.70^b^
10	3.46 ± 1.06^b,c^				
11	5.00 ± 1.07^d^				
12	6.06 ± 0.59^e^				
13	2.73 ± 1.33^a^				

All values are mean ± standard deviation. 1: NaNO_2_ (120 ppm); 2: NaNO_2_ (40 ppm); 3: No additive; 4: NaNO_2_ (40 ppm) + sodium hypophosphite (1000 ppm) + paprika extract (0.1% v/w); 5: NaNO_2_ (40 ppm) + sodium hypophosphite (1000 ppm) + cochineal powder (0.002% w/w); 6: sodium hypophosphite(3000 ppm) + paprika powder (1% w/w); 7: sodium hypophosphite (3000 ppm) + paprika extract (0.1% v/w); 8: butylated hydroxyl anisole (30 ppm); 9: NaNO_2_ (40 ppm) + butylated hydroxyl anisole (30 ppm); 10: paprika powder (0.1% w/w); 11: cochineal powder (0.02% w/w); 12: cochineal powder (0.015% w/w); 13: paprika extract (1.5% v/w).

^a–e^values within each column with different superscripts are significantly different (*P* < 0.05).

## Conclusions

To conclude, several nitrite-free and low-nitrite frankfurters consisting of cochineal and paprika as color agent, BHA as antioxidant, and SHP as antimicrobial agent were formulated in this study. The results indicate that the addition of these alternatives to nitrite had no effect on pH values and proximate analyses. Additionally, the results show that BHA is an effective antioxidant and prevents lipid oxidation in nitrite-free and low-nitrite samples at level of 30 ppm. SHP affected microbial growth in samples at level of 1000 ppm in low-nitrite product and 3000 ppm in nitrite-free product at both 4 and 10°C and retarded microbial spoilage in frankfurter. Also, SHP (1000 ppm in low-nitrite and 3000 ppm in nitrite-free frankfurter) inhibited the toxin production by *C. botulinum* at 25°C for at least 3 days. Addition of nitrite in lower concentration (40 ppm) showed that it is sufficient for producing a pink color in low-nitrite frankfurter, which was not significantly different (*P* > 0.05) from control TRT (120 ppm nitrite). However, 0.015% cochineal was required in nitrite-free product for producing a suitable pink color. Sensory evaluation results confirmed the color evaluation results. Sensory evaluation also showed that there was no significant difference between aroma and texture of various specimens, but the flavor of frankfurters containing 40 and 120 ppm nitrite had a high score in comparison with nitrite-free products. The data from this investigation support the use of cochineal as a colorant, SHP as an antibotulinal and antimicrobial agent, and BHA as an antioxidant in developing composite nitrite-free and low-nitrite frankfurter.
